# Nasal Reconstruction After Skin Cancer Excision: Clinical and Patient-Reported Outcomes from a Retrospective Study

**DOI:** 10.3390/jcm15062274

**Published:** 2026-03-17

**Authors:** Fabiana Battaglia, Michele Rosario Colonna, Simone Filistad, Roberta Giuffrida, Gabriele Delia

**Affiliations:** 1 Department of Plastic and Reconstructive Surgery, University Hospital of Messina “AOU Gaetano Martino”, 98125 Messina, Italy; mrcolonna1@gmail.com (M.R.C.); s.filistad93@gmail.com (S.F.); gabriele.delia@unime.it (G.D.); 2 Section of Dermatology, Department of Clinical and Experimental Medicine, University of Messina, 98125 Messina, Italy; roberta_giuffrida@hotmail.it

**Keywords:** nasal reconstruction, local flaps, non-melanoma skin cancer, patient-reported outcomes, aesthetic and functional results

## Abstract

**Background/Objectives**: Nasal reconstruction after non-melanoma skin cancer excision remains challenging due to the need to restore both nasal form and function while ensuring oncologic safety. Beyond surgical success, patient-reported outcomes are increasingly recognized as essential components of postoperative evaluation. The aim of this study was to retrospectively assess oncologic, surgical, and patient-reported outcomes in a real-world cohort of patients undergoing nasal reconstruction following skin cancer excision. **Methods**: A retrospective cohort study was conducted on 60 patients treated at the University Hospital “G. Martino” (Messina, Italy) between 2019 and 2022. Reconstructive techniques included direct closure, full-thickness skin grafts, local or regional flaps. Oncologic outcomes and postoperative complications were recorded during routine follow-up. Patient-reported outcomes were evaluated using a semi-structured PROM-derived questionnaire adapted from the FACE-Q Skin Cancer Module, NOSE, and SCaFF domains. Internal consistency of the questionnaire was assessed using Cronbach’s alpha. **Results**: Basal cell carcinoma was the most frequent diagnosis (55%), and the nasal ala, dorsum, and tip were the most commonly involved subunits. Local flaps were performed in 42% of cases. No histologically confirmed recurrences were observed in the flap-reconstructed subgroup during the available follow-up, whereas recurrences were observed in patients managed with non-flap reconstructive approaches. Postoperative complications were uncommon; however, one fatal infectious event occurred in a high-risk patient undergoing complex reconstruction for recurrent disease. The PROM-derived questionnaire demonstrated good internal consistency (Cronbach’s α = 0.82). Functional symptoms were rare, with 93% of patients reporting no snoring or nasal obstruction and 97% reporting no nasal voice alteration. Aesthetic satisfaction was rated as satisfactory or very satisfactory by 63% of patients, and social relationships were not affected in 85%. Patient-perceived recurrence risk (38%) exceeded histologically confirmed recurrence (15%). **Conclusions**: In this elderly real-world cohort, flap-based nasal reconstruction was associated with generally favorable patient-centered outcomes and low complication rates. The discrepancy between patient-perceived and confirmed recurrence highlights the role of oncologic anxiety. Prospective studies using fully validated PROMs are warranted to support standardized outcome comparison and guide clinical decision-making.

## 1. Introduction

Nasal reconstruction following oncologic excision is a complex endeavor that necessitates the restoration of both aesthetic appearance and functional integrity. The nose’s central position on the face makes it a focal point for facial harmony and individual identity, thereby amplifying the psychological impact of any deformity or scarring resulting from surgical interventions.

The subunit principle, introduced by Burget and Menick, emphasizes replacement of entire aesthetic subunits to optimize contour, scar camouflage, and long-term aesthetic integration in nasal reconstruction [1]. However, despite advancements in surgical techniques, there remains a paucity of comprehensive data evaluating long-term patient satisfaction and functional results post-reconstruction [2].

Nasal reconstruction following skin cancer excision encompasses a broad spectrum of reconstructive strategies, including primary closure, skin grafting, and local or regional flap reconstruction. The selection of the appropriate technique depends on multiple variables such as defect size, anatomical subunit involvement, patient comorbidities, and oncologic characteristics. Therefore, evaluating outcomes across heterogeneous reconstructive approaches may provide a more comprehensive and clinically meaningful understanding of postoperative results.

Patient-reported outcome measures (PROMs) have emerged as vital tools for evaluating reconstructive outcomes from the patient’s perspective in facial oncologic surgery [3,4].

Instruments such as the FACE-Q Skin Cancer Module have demonstrated acceptable psychometric properties in assessing satisfaction and quality of life following facial skin cancer reconstruction [5,6].

These tools have demonstrated reliability and validity in capturing patient experiences related to appearance, emotional well-being, and social interactions [7].

Recent studies applying validated PROM instruments in nasal oncologic reconstruction have demonstrated their utility in capturing aesthetic, functional, and psychosocial outcomes [6,8].

For instance, Theelen et al. conducted a cross-sectional cohort study assessing patient satisfaction following primary closure or second intention healing after conventional nasal skin cancer excision. The study found high levels of facial and scar satisfaction, low appearance-related distress, and minimal adverse effects in both groups, underscoring the value of PROMs in capturing nuanced patient experiences [9].

Moreover, systematic reviews have emphasized the need for standardized and validated PROMs in facial skin cancer reconstruction. Dobbs et al. identified several PROMs, including the FACE-Q and Skin Cancer Index, as having adequate psychometric properties for assessing outcomes in soft-tissue facial reconstruction. However, the study also noted variability in methodological quality and called for further validation to ensure these instruments effectively capture patient concerns [3,4].

In light of these considerations, our study aims to retrospectively evaluate clinical and patient-reported outcomes in a cohort of patients who underwent nasal reconstruction after non-melanoma skin cancer excision. This study was designed as a descriptive retrospective cohort analysis intended to provide a pragmatic overview of real-world reconstructive practice rather than a formal comparative study between specific techniques. We therefore report oncologic outcomes, complication profiles, and patient-reported measures across reconstructive strategies (direct closure, grafting, and flap-based approaches) to support patient-centered decision-making.

## 2. Materials and Methods

### 2.1. Ethical Approval and Study Design

A retrospective cohort study was conducted at the Plastic Surgery Unit of the University Hospital “G. Martino” in Messina, Italy, involving 60 patients who underwent nasal reconstruction following surgical excision of non-melanoma skin cancers between June 2019 and June 2022. The study was designed in compliance with the principles of the Declaration of Helsinki. All data were anonymized and derived from routine clinical practice.

Dermoscopic and videodermatoscopic examinations were performed exclusively as part of routine clinical assessment for the diagnosis and follow-up of cutaneous neoplasms, according to institutional standard-of-care protocols. No additional diagnostic procedures were introduced for research purposes.

### 2.2. Data Collection and Patient Cohort

Eligibility criteria included adult patients of either sex with histologically confirmed keratinocyte carcinomas (basal cell or squamous cell carcinoma) or premalignant nasal lesions requiring reconstructive procedures. Patients with melanoma, those lacking complete clinical documentation, or with unclear surgical history were excluded.

For each patient, data were collected on age at diagnosis, sex, residence, anatomical subunit involved, histopathological diagnosis, presence of ulceration and/or deep tissue invasion, type and date of intervention, and reconstructive technique adopted.

### 2.3. Surgical Techniques

Surgical approaches included direct closure, full-thickness skin grafts (from preauricular, retroauricular, or supraclavicular donor sites), cartilage grafts (septal or auricular), local or regional flaps (nasolabial, bilobed, glabellar, forehead, or V-Y flaps).

Non-surgical CO_2_ laser was not considered a reconstructive technique in the present study; two patients underwent an initial CO_2_ laser treatment attempt before definitive surgical management and were not analyzed as a separate subgroup.

Clinical photographs were obtained using standardized positioning (frontal, lateral, and oblique views when applicable), neutral background, and consistent lighting conditions to ensure reproducibility and editorial consistency.

Particular attention was paid to donor site management and flap inset geometry to minimize standing cone (“dog-ear”) deformities and prevent alar distortion. When designing advancement or rotation flaps, Burow’s triangles were strategically placed along relaxed skin tension lines or within natural creases (alar-facial sulcus or nasolabial fold) to redistribute tension away from free margins. In defects involving the alar rim or tip, tension vectors were oriented parallel to the alar margin to avoid vertical traction and secondary alar retraction. Conservative defatting and layered closure were performed to optimize contour and reduce trap-door effect.

Reconstructive decision-making followed a standardized algorithm routinely adopted in our unit, integrating defect size, anatomical subunit involvement, tissue depth, cartilage exposure, skin laxity, and patient-related factors (age, comorbidities, and anesthetic risk).

Direct closure was generally preferred for small, superficial defects with adequate surrounding laxity and without involvement of free margins. Full-thickness skin grafting was primarily selected for superficial defects not amenable to direct closure, particularly when a stable vascular bed was present and cartilage was not exposed (or after dermal template coverage when required).

Local or regional flaps were favored for larger defects, for lesions involving or crossing aesthetic subunits, for defects affecting free margins (e.g., alar rim, tip, columella), and in cases with cartilage exposure or greater depth requiring well-vascularized tissue to restore contour and function.

When flap reconstruction was indicated, flap selection was further tailored according to defect location, size, and involvement of specific nasal subunits. Bilobed flaps were generally preferred for small-to-moderate defects of the distal third of the nose (particularly the tip and supratip) requiring limited arc of rotation and good color match.

Nasolabial flaps were favored for alar and perialar defects requiring robust vascular supply and soft tissue bulk.

Rieger–Marchac (dorsal nasal) flaps were selected primarily for medium-sized defects of the nasal dorsum or proximal third, particularly when adjacent tissue laxity allowed advancement with preservation of contour.

Frontonasal or paramedian-type flaps were considered for larger or multi-subunit defects requiring greater tissue recruitment and structural support.

Final flap choice was individualized based on intraoperative assessment of tissue mobility, aesthetic subunit boundaries, and the need to maintain airway patency and contour symmetry.

The final reconstructive choice was confirmed intraoperatively after complete oncologic excision, prioritizing oncologic safety, preservation of nasal function, and aesthetic subunit reconstruction.

### 2.4. Postoperative Scar Assessment and PROM Evaluation

Follow-up was performed at 1, 3, 6, and 12 months postoperatively, and annually thereafter through outpatient visits or teleconsultations. Tumor recurrence was defined as histologically confirmed reappearance at the surgical site within the follow-up period.

The median follow-up duration was 12 months (range 6–36 months), based on scheduled clinical and/or histopathological surveillance.

In all 60 patients, a semi-structured questionnaire adapted from validated PROMs (FACE-Q Skin Cancer Module, NOSE, and SCaFF) was administered during follow-up visits. No preoperative PROM assessment was performed; therefore, patient-reported outcomes reflect postoperative perceptions without baseline comparison. The adaptation process included a preliminary pilot test conducted in 10 patients prior to full cohort administration, to ensure clarity and internal consistency. The questionnaire comprised ten items assessing aesthetic, functional, psychosocial, and oncologic domains.

The decision not to administer fully validated PROM instruments was primarily related to feasibility considerations, including consultation time constraints in an elderly population and the need for a concise instrument adaptable to routine outpatient practice.

The questionnaire was structured to evaluate several patient-centered outcomes, including scar quality, cutaneous retraction, nasal obstruction, nasal resonance, snoring, respiratory difficulty, social discomfort, aesthetic satisfaction, functional outcome, and recurrence awareness. Questionnaires were administered during routine follow-up visits by the treating surgical team.

Ratings were based on qualitative categories and Likert-type scales (Table 1).

Internal consistency was evaluated using Cronbach’s α, which showed satisfactory reliability (α = 0.82).

The questionnaire was conceived as a pragmatic, feasibility-oriented tool inspired by core domains of validated PROMs (FACE-Q, NOSE, and SCaFF), rather than as a formally validated instrument. It was developed to enable multidomain assessment within routine clinical follow-up visits, where time constraints and the absence of fully validated Italian-language versions of some instruments limited direct implementation of established PROMs.

The Likert-type scale used in the present study was not derived from a formally validated PROM instrument but was constructed to reflect core domains of established tools (FACE-Q, NOSE, and SCaFF) in a simplified format suitable for routine clinical practice.

### 2.5. Statistical Analysis

Two independent surgeons reviewed clinical documentation and questionnaire responses to ensure accuracy and consistency. Descriptive statistical analysis was performed to summarize demographic data, surgical techniques, and subjective outcome measures.

Given the heterogeneity of lesion characteristics, reconstructive modalities, and adjunctive procedures, the study was not designed nor powered for formal comparative statistical testing between specific techniques; therefore, results are presented primarily as descriptive and exploratory.

Recurrence rates and complication profiles were analyzed in relation to the reconstructive method employed.

All statistical analyses were conducted using IBM SPSS Statistics for Windows, version 28.0 (IBM Corp., Armonk, NY, USA). Results were expressed as frequencies, percentages, means, and standard deviations where appropriate.

## 3. Results

A total of 60 patients who underwent nasal reconstruction following oncologic excision were included in the present analysis. The cohort was composed of 42 males (70%) and 18 females (30%). The majority of patients resided in the city of Messina (50%) or its surrounding province (45%), while a small subset (5%) came from other regions of Sicily or from Calabria.

Patient ages ranged from 56 to 93 years. Specifically, 22 patients (37%) were younger than 75 years, 3 (5%) were exactly 75 years old, and 35 (58%) were older than 75 years.

Histopathological examination revealed a predominance of basal cell carcinomas (BCCs), accounting for 55% of all cases. This finding contrasts with existing literature, which typically reports a higher frequency of squamous cell carcinomas (SCCs) in the nasal pyramid. Among BCCs, the nodular subtype was the most frequently observed (25%). Actinic keratoses, considered precursors of cutaneous SCC (cSCC), were present in 23% of patients, and in some cases exhibited histological features suggestive of progression toward invasive carcinoma. Basosquamous carcinomas represented 12% of the cohort, while benign lesions such as hyperkeratotic warts and trichoepitheliomas were identified in 3% of cases.

Excised skin specimens measured between 0.5 and 3 cm in length in 85% of cases, and between 0 and 2 cm in width in 93% of cases. Histological assessment also included evaluation of dermal invasion depth, classified as one-third, one-half, two-thirds, or full-thickness involvement. Superficial ulceration was observed in 42% of lesions, a finding suggestive of delayed diagnosis and potentially increased risk for vascular invasion and neoplastic angiogenesis.

Anatomically, the nasal ala was the most frequently involved subunit (40%), followed by the nasal dorsum (35%) and nasal tip (22%). Less commonly affected regions included the lateral sidewalls (12%), nasolabial fold (12%), glabella (3%), and vestibular mucosa (2%). In a substantial number of patients, tumor extension across multiple nasal subunits was noted.

Reconstruction was categorized according to the primary skin coverage technique (direct closure, full-thickness skin grafting, or local flap reconstruction). Cartilage grafts and dermal substitutes were recorded as adjunctive procedures when applicable.

Direct closure represented the simplest reconstructive approach, employed in 30% of cases. Minor postoperative complications included wound dehiscence (11%) and late scarring, including hypertrophic or retracted scars. One patient (6%) developed a keloid scar.

Grafting was performed in 28% of patients. Among these, 18% received cartilage grafts harvested from the nasal septum or auricular concha, while 82% underwent full-thickness skin grafting. Donor sites included the supraclavicular region (55%), preauricular area (20%), and retroauricular area (10%). In 90% of grafting cases, Integra^®^ dermal regeneration template (Integra LifeSciences, Princeton, NJ, USA) was utilized, with a reported 100% engraftment rate. All grafts were secured with tie-over dressings.

**Figure 1:**
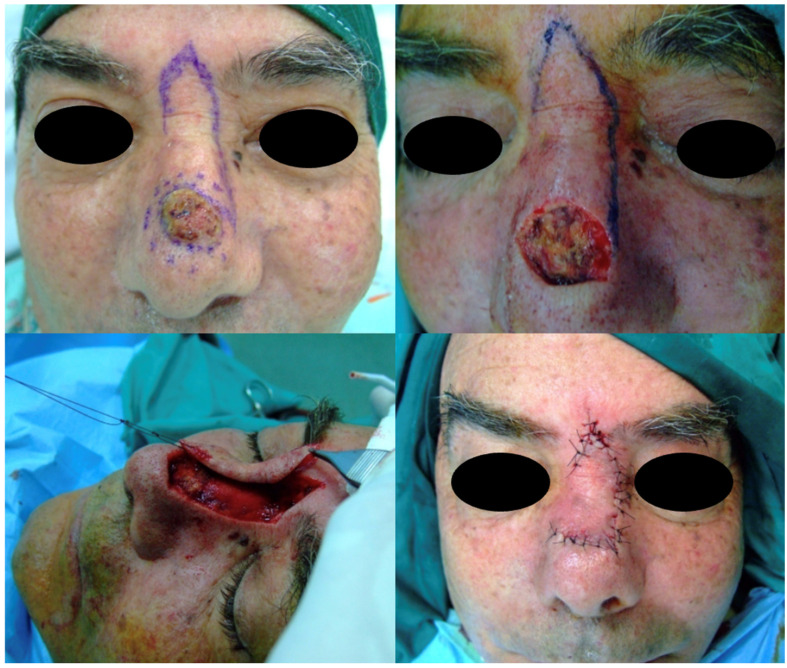
Reconstruction with Rieger–Marchac flap for dorsal nasal defect. The image shows the postoperative appearance after inset of the flap and suture. Note the alignment and contour matching of the nasal dorsum.

**Figure 2:**
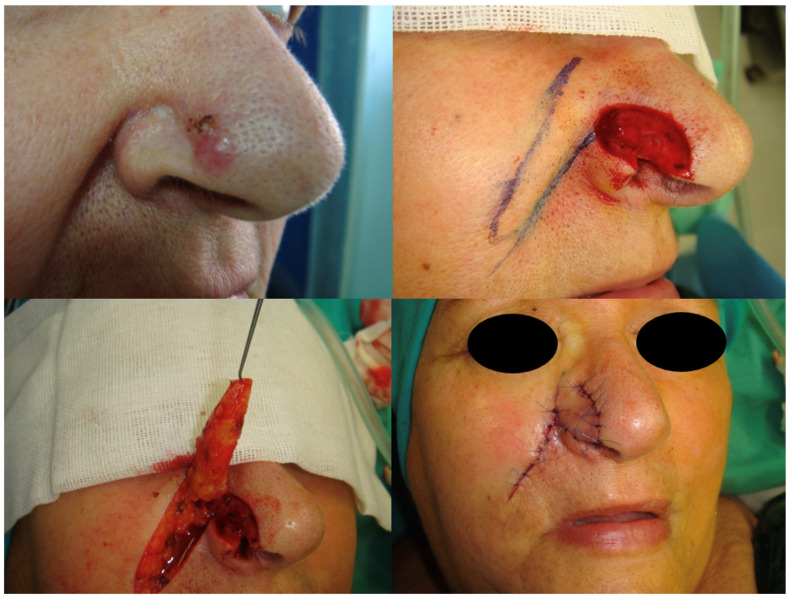
Lateral nasal ala reconstruction using a nasolabial flap. The surgical design and inset of the flap are depicted, highlighting the curvature and skin match in the perialar region.

**Figure 3:**
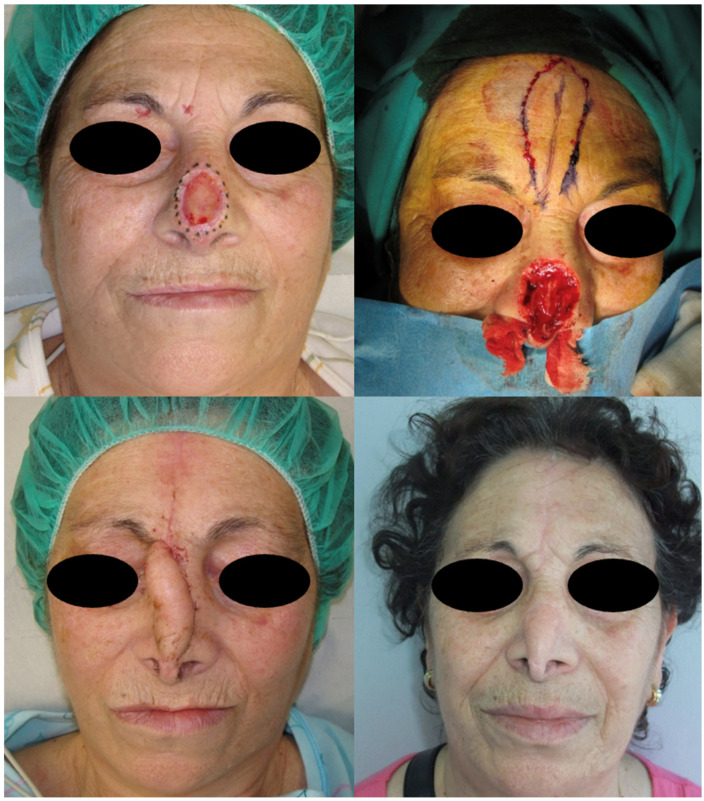
Frontonasal flap reconstruction for nasal tip and columella defects. The final aesthetic result is shown at 6-month follow-up, demonstrating symmetry, good color match, and minimal scarring.

Flap-based reconstruction was carried out in 42% of patients. Specific techniques included nasolabial flaps (29%), advancement flaps (20%), frontonasal flaps (16%), bilobed flaps (12%), glabellar or V-Y advancement flaps (8%), and Marchac transposition flaps (4%) (Figure 1, Figure 2 and Figure 3).

Two patients initially underwent a non-surgical CO_2_ laser treatment attempt prior to definitive surgical reconstruction; these cases were not analyzed as a separate subgroup due to the extremely small number.

Major postoperative events were uncommon; however, one sub-flap hematoma required surgical drainage, and one fatal infectious complication occurred in the context of complex reconstruction for recurrent disease, emphasizing that advanced age, comorbidity burden, and recurrent tumors may substantially increase perioperative risk. A detailed distribution of postoperative complications according to reconstructive technique is provided in Table 2.

Postoperative complications were uncommon across the cohort. Minor complications included two cases of wound dehiscence and one keloid scar following direct closure. Among patients undergoing flap reconstruction, one sub-flap hematoma required surgical drainage and one patient developed a severe postoperative infection that proved fatal in the context of complex reconstruction for recurrent disease.

Tumor recurrence occurred in a subset of patients during follow-up. Recurrences were observed among patients managed with less complex primary approaches and in cases previously treated or presenting with recurrent disease. Notably, no recurrence events were observed within the flap-reconstructed subgroup during the available follow-up; however, given the retrospective design and potential selection factors (lesion characteristics, margin status, and treatment history) this observation should be interpreted cautiously and considered hypothesis-generating rather than confirmatory.

All 60 patients completed a postoperative self-assessment questionnaire. The internal consistency of the adapted questionnaire was good (Cronbach’s α = 0.82). Patient-reported outcomes were stratified according to the primary reconstructive technique (direct closure, skin grafting, and flap-based reconstruction) (Table 3).

Among the 60 patients who completed the postoperative questionnaire, multiple domains were assessed, including aesthetic outcomes, functional symptoms, psychosocial impact, perceived risk of recurrence, and overall satisfaction.

With regard to aesthetic outcomes, 15% of patients reported no visible postoperative scarring, whereas mild, moderate, and severe scarring were reported by 45%, 22%, and 18% of patients, respectively, including cases of hypertrophic or keloid scars. Skin retraction was absent in 33% of patients, while mild, moderate, and severe degrees were reported in 27%, 22%, and 18% of cases, respectively.

Functional symptoms were generally uncommon. The majority of patients reported no postoperative snoring (93%), nasal obstruction (93%), or nasal voice alteration (97%). Mild functional symptoms were reported in a small minority of cases, and no moderate or severe functional impairment was observed.

From a psychosocial perspective, most patients (85%) reported no difficulties in social relationships following surgery. Mild social discomfort was reported by 3% of patients, while moderate difficulties were reported by 12%. No patients reported severe social impairment.

Regarding oncologic perception, 7% of patients reported no concern about recurrence, while 55% perceived recurrence as unlikely. Conversely, 18% considered recurrence likely and 20% considered it certain. Notably, patient-perceived recurrence exceeded the rate of histologically confirmed recurrence observed during follow-up.

Overall satisfaction was favourable. Aesthetic satisfaction was rated as satisfactory or very satisfactory by 63% of patients, while 15% reported dissatisfaction and 22% reported low satisfaction. Functional satisfaction was higher, with 73% of patients reporting satisfactory outcomes and 20% reporting very satisfactory outcomes; only 7% expressed low or unsatisfactory functional satisfaction.

A discrepancy was observed between objective oncologic outcomes and patient-perceived recurrence risk. A substantial proportion of patients reported moderate-to-high concern regarding recurrence despite the absence of histologic confirmation in most cases.

## 4. Discussion

Nasal reconstruction following oncologic excision is a demanding surgical endeavour, requiring the careful balance of three key objectives: oncologic safety, functional restoration, and aesthetic harmony. In our cohort, basal cell carcinoma (BCC) was the most frequent diagnosis (55%). While some studies have reported different distributions of keratinocyte carcinomas across anatomical sites and populations, our data are derived from a small, single-center series and should not be interpreted as evidence of an epidemiologic shift. Rather, this finding likely reflects local referral patterns, case mix, and the specific inclusion criteria of the present study [10,11].

A significant proportion of patients (23%) also presented with actinic keratoses, confirming the prominent role of chronic ultraviolet exposure as a carcinogenic factor [12]. Moreover, 42% of lesions showed superficial ulceration, which is recognized in the literature as a marker of tumor aggressiveness and a predictor of vascular invasion and local recurrence [13].

Beyond oncologic characteristics, an important contribution of the present study lies in the integration of patient-reported outcome domains into routine postoperative assessment. Rather than aiming to demonstrate the superiority of a specific reconstructive technique, our findings highlight the feasibility of a pragmatic, multidomain PROM-derived approach capable of capturing functional, aesthetic, psychosocial, and oncologic perception outcomes in a real-world clinical setting.

The choice of reconstructive strategy remains a key determinant of clinical outcomes.

Reconstructive strategies in nasal surgery range from direct closure and skin grafting to local and regional flaps. Each approach presents specific advantages and limitations in terms of vascular reliability, aesthetic integration, donor-site morbidity, and oncologic monitoring [14,15].These principles align with the subunit concept proposed by Burget and Menick, which advocates replacement of entire aesthetic subunits to achieve harmonious and durable results [1].

In our cohort, no histologically confirmed recurrences were observed among patients reconstructed with local flaps during the available follow-up. Given the retrospective design and potential selection bias, this observation should be interpreted cautiously and considered hypothesis-generating rather than confirmatory.

In contrast, recurrences were observed in patients managed with direct closure or full-thickness skin grafting. However, differences in recurrence distribution across reconstructive modalities should not be interpreted as evidence of a causal relationship between reconstruction type and oncologic control. Recurrence in keratinocyte carcinoma surgery is primarily influenced by adequacy of tumor excision and margin status rather than by the reconstructive technique itself [16,17]. Similarly, CO_2_ laser monotherapy, while less invasive, has demonstrated variable efficacy and a higher risk of incomplete tumor eradication when not combined with histologic margin assessment [18].

Fractional laser technologies have been reported to improve scar texture, pigmentation, and pliability through controlled dermal remodeling and stimulation of neocollagenesis and may represent a useful adjunct in postoperative scar modulation following reconstructive procedures [19,20].

Although the overall complication rate in our cohort was low, one fatal infectious complication occurred following flap reconstruction in the setting of recurrent disease.

This fatal event may be interpreted as a reminder that reconstructive success depends not only on surgical technique but also on appropriate perioperative risk stratification and surveillance, particularly in elderly patients with recurrent disease.

These patients represent a particularly vulnerable population, emphasizing the need for structured perioperative management and early detection of complications [13].

Patient-reported outcome measures provided valuable insight into subjective surgical success. Although the questionnaire used in this study was not formally validated, it was developed by adapting core domains from internationally recognized PROMs, including the FACE-Q, NOSE, and SCaFF instruments.

Recent studies have further emphasized the relevance of integrating both patient-reported and observer-based scar assessments following facial reconstruction. For example, a recent study on keystone flap reconstruction demonstrated the usefulness of combining patient and observer scar evaluation to better characterize aesthetic outcomes after facial reconstructive procedures [21].

Although the questionnaire demonstrated satisfactory internal consistency (Cronbach’s α = 0.82), it was not designed to replace formally validated PROM instruments. Rather, it should be interpreted as a feasibility-oriented, exploratory tool intended to capture key patient-centered domains within routine clinical practice. Future prospective studies should incorporate fully validated and culturally adapted PROM instruments to allow standardized outcome comparison.

Despite the objective presence of hypertrophic or retracted scars in a substantial proportion of patients, aesthetic satisfaction was reported by 63% of the cohort, while functional satisfaction reached 93%, consistent with previously published patient-centered outcomes in nasal oncologic reconstruction [5,6].

These findings suggest that functional restoration is often prioritized by patients over minor aesthetic imperfections, particularly in an elderly population.

Social reintegration after surgery was preserved in the vast majority of patients, with 85% reporting no interference in interpersonal relationships. These results are in line with those of Theelen et al., who demonstrated high satisfaction and minimal psychosocial burden following nasal skin cancer excision across different reconstructive strategies [9].

Interestingly, a notable discrepancy emerged between histologically confirmed recurrence (15%) and patient-perceived recurrence risk (38%).

This discrepancy may reflect underlying oncologic anxiety and highlights the importance of structured counseling and clear communication during postoperative follow-up.

This gap highlights the psychological vulnerability of patients undergoing facial oncologic procedures and suggests that oncologic reassurance is not automatically achieved by tumor clearance alone. Addressing oncologic anxiety through structured counseling and clear postoperative communication may therefore represent an essential component of comprehensive patient-centered care [22,23].

Several limitations must be acknowledged, including the retrospective single-center design, heterogeneity of lesion characteristics, and the use of a PROM-derived, non-validated questionnaire. Additionally, although follow-up was conducted up to 12 months in most patients, the median follow-up duration was 12 months (range 6–36 months). This duration may be insufficient to fully capture long-term aesthetic outcomes such as trap-door deformity, progressive scar maturation, or late contour irregularities, which can evolve beyond the first postoperative year. Longer prospective follow-up would allow more comprehensive assessment of late reconstructive sequelae. Additionally, the absence of preoperative patient-reported assessments precludes direct comparison between baseline and postoperative satisfaction levels. Furthermore, the adapted questionnaire was intended to assess feasibility and internal reliability rather than to serve as a fully validated PROM. Nevertheless, this approach enabled a multidimensional evaluation of outcomes that are often underreported in reconstructive series.

Overall, our findings suggest that simplified PROM-derived tools inspired by validated instruments can be effectively implemented in routine surgical practice, particularly when formal validated questionnaires are unavailable in the local language or clinical context. The absence of a fully validated instrument such as FACE-Q or SCAR-Q limits comparability with other published series and should be considered when interpreting patient-reported outcomes. Future prospective studies should incorporate fully validated PROMs such as FACE-Q and SCaFF [8,24], and further explore the relationship between objective surgical metrics, oncologic outcomes, and patient-reported quality of life.

## 5. Conclusions

Reconstruction of the nasal region following skin cancer excision represents a complex surgical challenge that requires careful integration of oncologic safety, functional restoration, and aesthetic outcome. In this retrospective cohort, heterogeneous reconstructive approaches were implemented in a real-world elderly population, achieving high functional satisfaction and acceptable aesthetic results, with a low overall complication rate.

Beyond technical outcomes, the integration of patient-reported outcome domains allowed for a more comprehensive evaluation of postoperative success. Functional satisfaction and social reintegration were largely preserved despite the frequent presence of visible scarring, underscoring the importance of assessing outcomes from the patient’s perspective.

Notably, the discrepancy between histologically confirmed recurrence and patient-perceived recurrence risk highlights the relevance of oncologic anxiety as an underrecognized determinant of postoperative satisfaction. Addressing patient understanding of recurrence risk through structured counseling and clear follow-up communication should therefore be considered an integral component of postoperative care.

Although limited by its retrospective design and the use of a PROM-derived, non-validated questionnaire, this study demonstrates the feasibility of implementing pragmatic patient-reported outcome assessments in routine clinical practice. Future prospective, multicenter studies incorporating validated instruments such as FACE-Q and SCaFF are warranted to enable standardized outcome comparison and to further refine patient-centered reconstructive algorithms.

## Figures and Tables

**Table 1:** Questionnaire items and domains used to assess patient-reported outcomes. Responses were recorded using a 4-point Likert scale (0 = none/unsatisfied to 3 = severe/very satisfied).

Item No.	Domain	Question	Response Scale (0–3)
1	Aesthetic	Visibility of the postoperative scar	0 = None, 3 = Severe
2	Aesthetic	Presence of skin retraction	0 = None, 3 = Severe
3	Functional	Postoperative nasal obstruction (difficulty breathing)	0 = None, 3 = Severe
4	Functional	Nasal voice presence	0 = None, 3 = Severe
5	Functional	Snoring	0 = None, 3 = Severe
6	Psychosocial	Impact on social relationships	0 = None, 3 = Severe
7	Oncologic	Subjective perception of tumor recurrence	0 = Not probable, 3 = Certain
8	Satisfaction	Aesthetic satisfaction	0 = Unsatisfied, 3 = Very Satisfied
9	Satisfaction	Functional satisfaction	0 = Unsatisfied, 3 = Very Satisfied
10	Other	Any additional postoperative complaint (free field)	Free text

**Table 2:** Postoperative complications according to reconstructive technique.

Complication	Direct Closure (*n* = 18)	Skin Graft (*n* = 17)	Local Flap (*n* = 25)
Wound dehiscence	2 (11%)	0	0
Hypertrophic scar	0	0	0
Keloid	1 (6%)	0	0
Sub-flap hematoma	0	0	1 (4%)
Major infection	0	0	1 (4%)
Revision surgery	0	0	0

**Table 3:** Patient-reported outcomes stratified by primary reconstructive technique (*n* = 60).

Outcome Metric	Direct Closure (*n* = 18)	Skin Graft (*n* = 17)	Local Flap (*n* = 25)
Visible scar (moderate-severe)	7 (39%)	7 (41%)	11 (44%)
Skin retraction (moderate-severe)	7 (39%)	7 (41%)	11 (44%)
Snoring	1 (6%)	1 (6%)	2 (8%)
Nasal voice	1 (6%)	0 (0%)	1 (4%)
Nasal obstruction	1 (6%)	1 (6%)	2 (8%)
Social relationship difficulties	2 (11%)	2 (12%)	3 (12%)
Tumor recurrence perception (moderate-severe)	7 (39%)	6 (35%)	10 (40%)
Aesthetic satisfaction (satisfied/very satisfied)	11 (61%)	11 (65%)	16 (64%)
Functional satisfaction (satisfied/very satisfied)	17 (94%)	16 (94%)	23 (92%)

## Data Availability

The data presented in this study are not publicly available due to privacy and ethical restrictions. Anonymized data may be made available from the corresponding author upon reasonable request and with permission of the Institutional Ethics Committee.

## Abbreviations

The following abbreviations are used in this manuscript:

BCC	Basal Cell Carcinoma
SCC	Squamous Cell Carcinoma
cSCC	Cutaneous Squamous Cell Carcinoma
PROMs	Patient-Reported Outcome Measures
FACE-Q	Facial Clinimetric Evaluation Questionnaire
NOSE	Nasal Obstruction Symptom Evaluation
SCaFF	Scar Cosmesis Assessment and Functional Score
CO_2_	Carbon Dioxide
IBM SPSS	International Business Machines Statistical Package for the Social Sciences
